# CT scan in the evaluation of pediatric abdominal trauma

**DOI:** 10.1590/0100-6991e-20223246-en

**Published:** 2022-11-23

**Authors:** ISABELLA PERIN, CAMILA ROGINSKI GUETTER, LÚCIO EDUARDO KLÜPPEL, CAMILA GIRARDI FACHIN, SILVANIA KLUG PIMENTEL TCBC-PR

**Affiliations:** 1 - Universidade Federal do Paraná - Curitiba - PR - Brasil; 2 - Johns Hopkins Bloomberg School of Public Health - Baltimore - Maryland - Estados Unidos; 3 - Hospital do Trabalhador, Radiologia - Curitiba - PR - Brasil; 4 - Hospital do Trabalhador, Cirurgia Geral - Curitiba - PR - Brasil

**Keywords:** Tomography, Radiation, Abdominal Injuries, Pediatrics, Tomografia, Radiação, Traumatismos Abdominais, Pediatria

## Abstract

**Objective:**

to assess the need of computed tomography (CT) for the definition of management in pediatric abdominal trauma.

**Methods:**

observational retrospective study with patients under 18 years old victims of blunt or penetrating abdominal trauma that underwent CT of the abdomen and pelvis at admission. We evaluated CT scan findings, indications and management. We calculated the sensitivity, specificity, positive predictive value and negative predictive value of clinical variables and energy of trauma for findings on CT.

**Results:**

among the 236 patients included in our study, 72% (n=170) did not present abnormal findings on CT. It was performed surgical treatment in 15% (n=10), conservative treatment in 54,5% (n=36) and 27% (n=18) did not receive treatment for abdominal injuries. In the assessment of CT indications, 28,8% (n=68) presented no justifications. In this group, 91% (n=62) did not show any abnormal findings. Among the six patients with positive findings, half were selected for conservative treatment, while the rest did not need any treatment for abdominal injuries. The presence of abdominal pain, hemodynamic alterations and high energy blunt trauma had low positive predictive values when isolated, whereas the negative predictive values were higher.

**Conclusion:**

although CT is necessary in some instances, there is a possible high number of exams that did not make any difference in the management of the pediatric population.

## RESUMO

**Introdução:**

avaliar a necessidade da tomografia computadorizada (TC) para definição de condutas em trauma abdominal pediátrico.

**Métodos:**

estudo observacional retrospectivo com pacientes menores de 18 anos vítimas de trauma abdominal contuso ou penetrante e que realizaram TC de abdome e pelve na admissão. Avaliou-se achados das tomografias, condutas e justificativas para indicação da TC. Foram calculados sensibilidade, especificidade, valor preditivo positivo e valor preditivo negativo de fatores clínicos e energia do trauma para alterações na TC.

**Resultados:**

dentre os 236 pacientes incluídos, 72% (n=170) não apresentaram alterações na TC. Foi realizado tratamento cirúrgico em 15% (n=10), tratamento conservador em 54,5% (n=36) e 27% (n=18) não receberam tratamento por lesões abdominais. Na avaliação das indicações de TC, 28,8% (n=68) não apresentavam nenhuma justificativa, sendo que nesse grupo 91% (n=62) resultaram em ausência de achados. Dentre os seis pacientes com achados positivos, metade recebeu tratamento conservador, enquanto o restante não necessitou de tratamento por lesões abdominais. A presença de dor abdominal, alteração hemodinâmica e trauma contuso de alta energia apresentaram baixos valores preditivos positivos de forma isolada, enquanto os valores preditivos negativos foram mais altos.

**Conclusão:**

apesar de a TC ser necessária e justificável em alguns casos, há um possível excesso de tomografias dispensáveis para definição de condutas em população pediátrica.

## INTRODUCTION

Due to its high sensitivity, Computed tomography (CT) is considered the gold standard for the evaluation of suspected intra-abdominal injuries after blunt trauma in adults^1^ and has been shown to be essential to prevent injuries from going unnoticed^2^. In addition, the wide use of this exam is related to numerous benefits, such as speed, accessibility, and high level of anatomical details provided by the image^3^ .

However, doses of ionizing radiation brought by CT can damage DNA, increasing the risk of cancer throughout life^3^ ^,^ ^5^ . In this sense, children are at greater risk, since they have greater radiosensitivity compared to adults, in addition to a longer life expectancy after exposure to radiation^3^. Pearce et al. demonstrated a two- to three-fold increase in the incidence of leukemia and brain tumors in individuals exposed to radiation in childhood^4^. Mathews et al. demonstrated a 24% increase in the global incidence of cancer in individuals exposed to radiation by CT, especially when exposure occurred at younger ages^5^ . Miglioretti et al. estimated that one year of pediatric CT in the United States could induce more than 4,800 future neoplasms^6^. In this sense, campaigns such as “Image Gently”^7^ and “As Low As Reasonably Achievable (ALARA)”^8^ have contributed to raising awareness of the risks associated with this test, as well as to developing protocols and recommendations for reducing radiation exposure in children.

In general, there are two strategies for controlling the exposure of children to radiation: one is to limit CT orders only to when there are justifiable indications such as those approved in protocols, and the other is through a technical adjustment in CT scanners to reduce radiation dose^9^. Multiphase contrast-enhanced CT generates two to four times more exposure to ionizing radiation^10^. Some of these strategies are used in our service. When evaluating children, CT scanners are programmed according to a pediatric protocol, which reduces radiation to the minimum possible for diagnosis. All patients undergoing CT receive contrast injection. However, unlike adults, pediatric contrast-enhanced tomography is performed in a single phase, in which two injections of contrast are performed with a time interval between them, to highlight the arterial and venous phases in the same image.

There are data indicating that children with minimal lesions are frequently submitted to CT, which will hardly alter the treatment approach^11^. A better selection of candidates for CT, especially in the presence of minimal lesions, could help achieve a balance between the risks and benefits of this exam.

The objective of this study is to evaluate the need for computerized tomography for conduct definition, considering the reasons used by the attending physician in different situations of pediatric abdominal trauma. We also evaluated sensitivity, specificity, and predictive values of clinical parameters and trauma mechanism in predicting CT findings.

## METHODS

This is a retrospective, cross-sectional, observational study carried out at a referral center for trauma care in Curitiba, Paraná State, Brazil, and its metropolitan region. The study was approved by the Ethics Committee under number 18809419.2.0000.5225.

We included patients under 18 years of age who had suffered blunt or penetrating abdominal trauma and underwent abdominal and pelvic CT on admission. We analyzed all records of pediatric abdominal and pelvic CT scans requested between September 2017 and September 2019. We excluded patients undergoing CT who were not trauma victims, as well as those whose medical records did not have all the data necessary for the analysis.

The following were considered positive findings on CT of the abdomen and pelvis: penetration of the cavity by a penetrating wound, solid viscera injuries, hematomas, pneumoperitoneum, free fluid in the abdominal cavity, and pelvic and lumbosacral spine fractures. Patient management was divided into 1) surgical treatment, 2) conservative treatment, and 3) absence of treatment for abdominal injuries, including patients without abdominal alterations on CT, those who were discharged without the need for hospitalization for observation, or who had only extra-abdominal injuries such as fractures, chest injuries, and traumatic brain injury (TBI). The reasons for CT indication were grouped into: A) trauma mechanism, including penetrating trauma and high-energy blunt trauma, B) presence of abdominal pain, and C) hemodynamic alteration. Patients with normal heart rate and blood pressure were considered hemodynamically stable. In case of hemodynamic instability, when the patients presented only tachycardia, they underwent CT after hydration with crystalloids, whereas hypotensive patients required stabilization with blood products transfusion before the exam.

We analyzed the collected data using the statistical software STATA, version 14^12^. For the descriptive analysis, we expressed measures of central tendency and dispersion as means and standard deviation (mean ± SD) for continuous variables with normal distribution. Categorical variables were expressed as absolute and relative frequencies. For inferential statistical analysis, we performed unpaired analyzes using the Student’s t test for continuous dependent variables and the Fisher’s exact test for binary or categorical dependent variables. We calculated sensitivity, specificity, positive predictive value (PPV), and negative predictive value (NPV) of clinical factors and trauma mechanism to identify changes on computed tomography. We also carried out a simple logistic regression to compare clinical factors and trauma mechanism (predictor variables) and the presence of alterations on computed tomography (outcome variable). This was presented with Odds Ratio and Confidence Interval. We considered a significance level of 5%.

## RESULTS

We evaluated 315 CT scans, of which we excluded 79, 53 because they were not from trauma patients and 26 whose medical records did not have enough data for analysis. Among the 236 patients included in the study, 66.5% (n=157) were male. Age ranged from nine months to 17 years, with a mean of 11.83 ± 4.95 years. Traumas were blunt in 86% (n=203) and penetrating in 14% (n=33). Among blunt traumas, 43% (n=87) were classified as high-energy, as they presented features such as vehicle ejection, roll over, absence of a seat belt, speed above 32km/h, falls greater than three meters, occurrence of death in the same scene, and reports of high energy described in the medical record. Twelve patients (5%) died, ten due to severe TBI, one due to spinal cord injury associated with infection, and one due to hemodynamic instability. Six patients (2.5%) underwent measurement of aspartate aminotransferase and pancreatic enzymes before CT.

There were positive findings on tomography in 28% (n=66) of the patients, while 72% (n=170) of the exams showed no alterations. When evaluating only the CT scans with positive findings, 15% (n=10) were submitted to surgical treatment, 54.5% (n=36) to conservative treatment, 27% (n=18) did not receive any treatment for abdominal injuries, and 3% (n=2) died before adoption of procedures for abdominal injuries. When evaluating all the patients studied, 95% (n=224) did not undergo surgical treatment after the CT scan, receiving only conservative treatment or not receiving abdominal treatment due to the absence of lesions. Among those undergoing conservative treatment, 33 were successful and three required surgical treatment during hospitalization. Among the 12 patients who underwent surgical treatment, 70% (n=7) had suspected hollow viscus injury due to the presence of pneumoperitoneum at CT.

Focused Assessment with Sonography for Trauma (FAST) was performed in 32.6% (n=77) of patients prior to CT. Among them, only 10.4% (n=8) had a positive result, and all of them also had a CT with positive findings. Among the 69 patients with negative FAST, 11 displayed findings of free fluid or viscera injury at CT, and two underwent surgical treatment.

In the 66 CT scans with positive findings, 51.5% (n=34) had a finding suggestive of solid viscus injury, 10.6% (n=7) pneumoperitoneum, 7.5% (n=5) only a small amount of free fluid, 16.6% (n=11) only pelvic fracture, 7.5% (n=5) only lumbosacral spine fracture, and 4.5% (n=3) only hematoma, one located in the adrenal and two in the pelvis. One CT scan showed penetration of the cavity by a stab wound, without organ damage.

Regarding the reasons for CT indication, 28.8% (n=68) had no reason, 9.7% (n=23) only abdominal pain, 8% (n=19) only hemodynamic changes, 20.7% (n=49) only high-energy mechanism, 27% (n=64) had two of the three previously mentioned reasons, while 5.5% (n=13) had the three simultaneously. Among the indication hemodynamic alteration, 80.5% (n=54) had only tachycardia.

When comparing the indications with the presence or absence of CT findings, we found that in the absence of any reason, 91% (n=61) of CTs had no alterations. In the presence of abdominal pain, hemodynamic alteration, and high-energy mechanism simultaneously, 84.6% (n=11) of CT had positive findings. This difference was statistically significant (p<0.001) (Table 1).

**Table 1:** Results of abdominal CT scans according to indication.

Indication	Negative CT	Positive CT
No indication	62 (91.1%)	6 (8.8%)
Abdominal pain	13 (56.5%)	10 (43.4%)
Hemodynamics	13 (68.4%)	6 (31.5%)
Mechanism	39 (79.5%)	10 (20.4%)
Abdominal pain + hemodynamics	1 (16.6%)	5 (83.3%)
Abdominal pain + mechanism	16 (55.1%)	13 (44.8%)
Hemodynamics + mechanism	24 (82.7%)	5 (17.2%)
Abdominal pain + hemodynamics + mechanism	2 (15.3%)	11 (84.6%)

Among the six altered CT scans in patients with no indication, three had only pelvic fractures and were treated only by orthopedics. One had only a small amount of free fluid and two showed liver contusion, all of which resulted in conservative treatment. Two surgeries were performed in patients with normal CT, one for removal of a penetrating object without penetration of the cavity and one due to trauma in the anal region. Half of the surgeries were performed on patients with penetrating trauma, so that 3% (n=6) of blunt trauma and 18% (n=6) of penetrating trauma resulted in surgical treatment. The 82% (n=27) of penetrating trauma that did not undergo surgical treatment had injuries without penetration of the cavity, including projectiles lodged in the pelvis or subcutaneously.

To assess the relationship between clinical parameters and trauma energy in the identification of CT alterations, those in which there was a certain indication for CT and the test results were positive were considered true positives. The true negatives were those in which there was an absence of a certain indication and the CT showed no alterations. The three parameters evaluated showed low values of sensitivity and PPV. Abdominal pain was the single factor with the highest specificity and NPV, with values of 81.2% and 83.6%, respectively (Table 2).

**Table 2:** List of clinical parameters and trauma energy for identification of changes in abdominal tomography.

Indication	Sensitivity	Specificity	PPV*	NPV**	OR***	95% CI****
1) Abdominal pain	59.1%	81.2%	54.9%	83.6%	6.23	3.35 - 11.60
2) Hemodynamic change	40.9%	76.5%	40.3%	76.9%	2.25	1.23 - 4.11
3) High energy (blunt trauma)	50.9%	60.1%	32.2%	76.7%	1.56	0.84 - 2.90
4) Abdominal pain + hemodynamic change	7.6%	99.4%	83.3%	73.5%	13.85	2.09 - δ
5) Abdominal pain + high energy	19.7%	90.6%	44.8%	74.4%	2.36	1.08 - 5.17
6) Hemodynamic change + high energy	7.6%	85.9%	17.2%	70.5%	2.36	0.19 - 1.33
7) Abdominal pain + hemodynamic change + high energy	16.7%	98.8%	84.6%	75.3%	16.80	4.02 - δ

*Positive Predictive Value; **Negative Predictive Value; ***Odds Ratio; ****95% confidence interval; *δ*: these higher confidence interval values could not be calculated correctly due to the low power of analysis in these subgroups (small number of individuals). The analysis of these confidence interval values and their respective Odds Ratio should take this limitation into account.

## DISCUSSION

The results demonstrated many CT scans that showed no changes, totaling 170 exams (72%) with negative findings. Among the altered CT scans, most resulted in conservative treatment (54.5%). When we evaluated only blunt trauma, only 3% (n=6) resulted in surgical treatment. In addition, 51.5% (n=34) of the altered exams showed lesions in solid viscera, while only 10.6% (n=7) had pneumoperitoneum, suggesting a hollow viscus lesion. In view of this, the question arises whether CT was essential for the choice between conservative or surgical management in these patients. In the literature, there are doubts as to whether tomography in pediatric patients is essential for establishing criteria for conservative treatment or for predicting the results of a non-operative approach^10^ . In most cases, the combination of ultrasound and serial abdominal physical examinations is sufficient to identify high-grade lesions^13^. In addition, conservative treatment of solid viscera lesions, even higher-grade ones, has proven to be a safe approach^14^, as long as the patient is hemodynamically stable^10^. A possible reason for the wide use of CT could be the fear of not diagnosing hollow viscus injuries that require surgical treatment. However, the combination of the limited sensitivity of CT in the identification of intestinal lesions with data that point to serial abdominal examinations as the most sensitive indicator of occult intestinal lesions^15^ should provide the physician with confidence that CT is best used in those patients with repeated altered physical examinations associated with laboratory alterations^16^ ^,^ ^17^.

When all patients were evaluated, 28.8% (n=68) did not have any of the three indications for CT, and 91% (n=62) of these exams resulted in no findings. Although eight CT scans showed alterations, all resulted in conservative treatment, and three involved only orthopedic treatment. In this group of patients, the need for CT to define conducts could be questioned, since the absence of both clinical alterations, such as abdominal pain and hemodynamic instability, and the mechanism of penetrating trauma or high-energy blunt trauma, would help in the identification of patients who can be spared the radiation of a CT scan in favor of a period of observation and laboratory testing. The literature shows similar data. Evaluating the performance of CT in pediatric patients, Streck et al. showed that 17% of the CT scans performed were in patients with very low risk of intra-abdominal injury^18^. In Holmes et al., 23% of CT scans were performed in children with very low risk of intra-abdominal injury that would require acute intervention^19^.

Several studies suggest that the combination of tools available in trauma centers, such as FAST and laboratory tests, could be used to stratify the risk of intra-abdominal injury in children victims of blunt trauma^20^ ^,^ ^21^. Prediction protocols were developed to try to identify more precisely in which cases CT could be avoided due to their very low risk of intra-abdominal injury after blunt trauma. One of the most recent was described by Streck et al.^18^ and consists of the evaluation of five parameters: aspartate aminotransferase levels >200U/L, abnormal abdominal physical examination, abnormal chest X ray, report of abdominal pain, and alteration of pancreatic enzymes. The patient without any of these factors is classified as very low risk of intra-abdominal injury. The protocol had a NPV of 99.4%^18^ and has already been validated in another recent study^22^. However, the acceptance of this type of guideline in different trauma centers is variable^23^, demonstrating difficulty in achieving universal application. One of the reasons could be the fact that several services do not frequently perform laboratory tests before CT at admission. In our study, only six patients (2.5%) underwent measurement of aspartate aminotransferase and pancreatic enzymes before the scan.

The three factors evaluated in our study (abdominal pain, hemodynamic stability, and trauma mechanism) are not sufficient for the application of validated prediction protocols such as the one by Streck et al., which also includes findings of complementary exams that are not carried out in our country due to unavailability or cost. Nonetheless, only with these three factors, easily obtainable in any service, it was possible to form a group containing 28.8% of patients who would benefit from an observation period with serial physical examinations and laboratory tests before the decision to perform CT. In addition, our results show the relevance of clinical history and physical examination, since the concomitant presence of abdominal pain, hemodynamic alteration, and high-energy mechanism brings an approximately 17 times greater chance (OR=16.80) of positive findings on tomography, in addition to a NPV of 75.3%.

The presence of abdominal pain, hemodynamic alteration, and high-energy blunt trauma alone had low positive predictive value and high negative predictive value. These results can be complemented by data from another study in which the physical examination findings showed a NPV of 98% for the need of operative treatment in children victims of vehicle collision trauma^24^. In addition, when comparing the isolated presence of each of the three indications, abdominal pain alone resulted in the highest number of CT scans with positive findings (43.4%). Abdominal pain also displayed the highest PPV (54.9%) and NPV (83.6%) compared to other indications alone. When the findings of abdominal pain and hemodynamic changes were simultaneously evaluated, the PPV increased to 83.3%. Along the same lines, another study identified that the most significant predictor of intra-abdominal injury requiring intervention was abnormal abdominal physical examination^18^.

This study has limitations, being retrospective and containing data from only one service, with a small number of patients evaluated. However, few previous studies have quantified imaging tests in children with no or minimal lesions^11^, resulting in a possible excessive indication of CT scans in pediatric patients.

Our study suggests that, despite being necessary and justifiable in some cases, there is a possible excess of unnecessary CT scans to define conducts in the pediatric population when in the absence of indications to justify the exam, resulting in reports with no changes or with minimal injuries. Furthermore, it shows low sensitivity and positive predictive values of findings such as abdominal pain, hemodynamic changes, and high-energy blunt trauma in isolation, but with greater relevance in the identification of positive findings when found simultaneously. The specificity and negative predictive values of these parameters were higher, even in isolation. The data found reinforce the need for studies with a greater number of patients, considering the specific conditions of our country, where often due to overcrowding and lack of physical structure and specific care for the pediatric population, one cannot easily apply protocols established in other countries to minimize the use of CT in children.
